# In‐depth proteomics characterization of ∆Np73 effectors identifies key proteins with diagnostic potential implicated in lymphangiogenesis, vasculogenesis and metastasis in colorectal cancer

**DOI:** 10.1002/1878-0261.13228

**Published:** 2022-06-07

**Authors:** María Garranzo‐Asensio, Javier Rodríguez‐Cobos, Coral San Millán, Carmen Poves, María Jesús Fernández‐Aceñero, Daniel Pastor‐Morate, David Viñal, Ana Montero‐Calle, Guillermo Solís‐Fernández, María‐Ángeles Ceron, Manuel Gámez‐Chiachio, Nuria Rodríguez, Ana Guzmán‐Aránguez, Rodrigo Barderas, Gemma Domínguez

**Affiliations:** ^1^ Chronic Disease Programme (UFIEC) Instituto de Salud Carlos III Madrid Spain; ^2^ Departamento de Bioquímica, Facultad de Medicina Instituto de Investigaciones Biomédicas “Alberto Sols”, CSIC‐UAM, IdiPAZ Madrid Spain; ^3^ Gastroenterology Unit Hospital Universitario Clínico San Carlos Madrid Spain; ^4^ Surgical Pathology Department Hospital Universitario Clínico San Carlos Madrid Spain; ^5^ Medical Oncology Department Hospital Universitario La Paz Madrid Spain; ^6^ Departamento de Bioquímica y Biología Molecular, Facultad de Óptica y Optometría Universidad Complutense de Madrid Spain

**Keywords:** ∆Np73 effectors, colorectal cancer, in‐depth proteomics, lymphangiogenesis, secretome

## Abstract

Colorectal cancer (CRC) is the third most common cancer and the second leading cause of cancer‐related death worldwide. Alterations in proteins of the p53‐family are a common event in CRC. ΔNp73, a p53‐family member, shows oncogenic properties and its effectors are largely unknown. We performed an in‐depth proteomics characterization of transcriptional control by ∆Np73 of the secretome of human colon cancer cells and validated its clinical potential. The secretome was analyzed using high‐density antibody microarrays and stable isotopic metabolic labeling. Validation was performed by semiquantitative PCR, ELISA, dot‐blot and western blot analysis. Evaluation of selected effectors was carried out using 60 plasma samples from CRC patients, individuals carrying premalignant colorectal lesions and colonoscopy‐negative controls. In total, 51 dysregulated proteins were observed showing at least 1.5‐foldchange in expression. We found an important association between the overexpression of ∆Np73 and effectors related to lymphangiogenesis, vasculogenesis and metastasis, such as brain‐derived neurotrophic factor (BDNF) and the putative aminoacyl tRNA synthase complex‐interacting multifunctional protein 1 (EMAP‐II)–vascular endothelial growth factor C–vascular endothelial growth factor receptor 3 axis. We further demonstrated the usefulness of BDNF as a potential CRC biomarker able to discriminate between CRC patients and premalignant individuals from controls with high sensitivity and specificity.

## Graphical abstract

High‐density antibody microarrays and stable isotopic metabolic labeling allowed the identification of secreted dysregulated effectors by the ectopic ∆Np73 expression in HCT116 colorectal cancer (CRC) cells. After validating the dysregulation of the effectors by orthogonal techniques, an important association with lymphangiogenesis, vasculogenesis and CRC metastasis was found. Brain‐derived neurotrophic factor was validated by ELISA as potential biomarker of CRC.

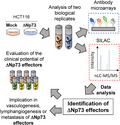

**Abbreviations**
ACN: acetonitrile
ATCC: American Type Culture Collection
AUC: area under the curve
BDNF: brain‐derived neurotrophic factor
CRC: colorectal cancer
DMEM: Dulbecco's modified Eagle's medium
ECM: endothelial cell medium
EMAP‐II: endothelial‐monocyte‐activating polypeptide II
GFR: growth factor reduced
HLEC: human lymphatic endothelial cells
HUVEC: human umbilical vein endothelial cells
ISCIII: Instituto de Salud Carlos III
MS: mass spectrometry
MTT: 3‐(4,5‐dimethylthiazol‐2‐yl)‐2,5‐diphenyl‐2H‐tetrazolium bromide
P/S: penicillin/streptomycin
qPCR: quantitative PCR
ROC: receiver operating characteristic
RT‐PCR: reverse transcriptase‐PCR
SDHA: succinate dehydrogenase complex subunit A
SILAC: stable isotopic labeling with amino acids in cell culture
siRNA: small interfering RNA
Trp: tryptophan
VEGFC: vascular endothelial growth factor C
VEGFR3: vascular endothelial growth factor receptor 3
WB: western blot

## 1 Introduction

Colorectal cancer (CRC) is the second most common cancer in Europe and the second leading cause of cancer‐related death following lung cancer. CRC appears sporadically with only a small percentage of the cases due to inherited predisposition [1]. Events leading to CRC development are driven by cumulative genetic alterations produced during decades, which induce a complex cascade of changes in the expression of transcription factors, cell signaling and cell–cell adhesion molecules, among others, that induce cancer cells to massively proliferate and acquire metastatic properties [2].

The *p53* family member, *TP73*, is translated into different isoforms [3]. *TP53* and *TP73* share key structural domains and functions [4], although their roles in tumorigenesis differ. Both genes are activated after DNA damage, triggering cell‐cycle arrest and cell death. Full length TAp73 isoforms have tumor‐suppressor potential [5], whereas ΔTAp73 variants lacking all or part of the transactivation domain in the amino terminal region due to alternative splicing and their transcription from a second promoter, show oncogenic properties [6].

Among the ΔTAp73 variants, ∆Np73, an isoform transcribed from the second alternative promoter, is known for its important role in cancer development, whose significant overexpression has been reported in most human cancers [1, 3]. Among other characteristics, ∆Np73 has been shown to have oncogenic properties and its up‐regulation is associated with shorter survival rates in different cancer types, including CRC [1, 3]. However, unlike other members of the p53‐family, effectors or downstream modulators of ∆Np73 are barely described at transcriptomic and/or proteomic level. Since cancer cells secrete proteins or protein fragments to different body fluids that can be used as biomarkers, the secretome of cancer cells constitutes a rich source of information for the identification of such biomarkers and for the characterization of altered molecules in the pathology [7].

In this context, we here performed an in‐depth proteomics characterization of the transcriptional control by ∆Np73 of the secretome of human stably transfected ∆Np73 and mock colon carcinoma HCT116 cells by using high‐density antibody microarrays and stable isotopic labeling with amino acid in cell culture (SILAC) to survey for altered pathways and modulated proteins due to ∆Np73 overexpression. Through the complementary combination of both proteomic methodologies, we identified a total of 51 dysregulated proteins that showed ≥ 1.5 or ≤ 0.67 fold‐change in the conditioned medium. Verification of the protein alterations by ∆Np73 was performed by semiquantitative PCR, ELISA, dot‐blot, and western blot (WB) on HCT116 and HCT116 p53^−/−^ cells to demonstrate that the presence of p53 was not affecting protein alterations by ∆Np73. Moreover, an evaluation of selected identified proteins and their possible biomarker value in plasma from CRC patients, colorectal individuals carrying premalignant lesions and colonoscopy‐negative controls was carried out. Among others, we found an important association between the overexpression of ∆Np73 and the VEGFC‐EMAP‐II‐VEGFR3 axis and the brain‐derived neurotrophic factor (BDNF) regulation of lymphangiogenesis, vasculogenesis and metastasis, and further demonstrated the usefulness of BDNF as a potential CRC biomarker able to discriminate between CRC and non‐CRC patients with high sensitivity and specificity.

## 2 Materials and methods

### 2.1 Cell lines, culture conditions and stable transfection of the HCT116 colon cancer cell line

The colon cancer cell line HCT116 was obtained from the American Type Culture Collection (ATCC, Manassas, VA, USA). HCT116 p53^−/−^ cells were obtained from Horizon Discovery (Waterbeach, UK). Human umbilical vein endothelial cells (HUVEC) and human lymphatic endothelial cells (HLEC) were purchased from Innoprot (#P10961; #P10571, Bizkaia, Spain). HCT116 cells were cultured in Dulbecco's modified Eagle's medium (DMEM; Corning #10‐013‐CVR, New York, NY, USA) supplemented with 10% heat‐inactivated FBS (Corning #35‐079‐CV), 2 mm l‐glutamine (Gibco; #25030‐081, Waltham, MA, USA), 1% penicillin/streptomycin solution (P/S; Corning #30‐009‐CI) and amphotericin B (0.25 μg·mL^−1^; Corning #30‐003‐*CF*). HUVEC cells were cultured in endothelial cell medium (ECM; Innoprot #P60104) supplemented with 5% heat‐inactivated FBS, 1% endothelial cell growth supplement and 1% P/S. HLEC cells were cultured in fibronectin‐coated plates and ECM medium supplemented as described below for HUVEC cells. HUVEC and HLEC cells were subjected to experimental procedures within passages 2–6.

HCT116 and HCT116 p53^−/−^ cells were stably transfected with the constructs pEF1α‐IRES‐GFP (empty vector –Mock control‐) or pEF1α‐ΔNp73β‐IRES‐GFP (ΔNp73 vector) plasmids (kindly provided by Dr Marín and Dr Marqués, Instituto de Biomedicina, Universidad de León, Spain) using Lipofectamine 2000 (Thermo Fisher Scientific, Waltham, MA, USA), according to the manufacturer's instructions. ΔNp73 overexpressing and Mock control cells were selected by cell sorting using GFP as selection marker.

### 2.2 Antibody microarrays, and SILAC proteomics approaches

#### 2.2.1 Antibody microarrays, sample labeling and antibody microarray screening

RayBio Label‐Based (L‐series) Human Antibody Arrays 493, consisting of two equal subarrays containing 493 unique antibodies, positive and negative controls spotted in duplicate, for the simultaneous detection of multiple cytokines, chemokines, adipokines, growth factors, angiogenic factors, proteases, soluble receptors, and soluble adhesion molecules, were obtained from RaybioTech. Microarrays were probed according to the manufacturer's instructions using biotin‐labeled samples followed by the incubation with streptavidin‐Cy3.

HCT116‐∆Np73 or control cells were grown and maintained in DMEM supplemented with 10% dialyzed FBS, 100 units·mL^−1^ of penicillin/streptomycin at 37 °C in 5% CO_2_. For preparation of conditioned medium, 5 × 10^6^ HCT116‐∆Np73 or control cells were seeded to achieve confluence after 24 h. Then, cells were washed with PBS, incubated with serum‐free medium for 1 h, washed again with PBS, and incubated for 48 h in serum‐free DMEM supplemented with antibiotics. Cell viability, determined with 0.4% trypan blue solution (Invitrogen, Waltham, MA, USA), was higher than 95%. The conditioned medium was centrifuged at 1500 ***g*** to remove cell debris, and protein concentration was measured by the tryptophan (Trp) method as described [8, 9], and stored at −80 °C until use. For incubation of RayBio Label‐Based (L‐series) Human Antibody Arrays 493, samples corresponding to two different biological replicates labeled with biotin were separately incubated on two arrays containing two equal subarrays. For biotin labeling, 30 μg of conditioned medium were incubated during 30 min at room temperature with gentle shaking with labeling reagent solution, as previously done [10]. The reaction was stopped with 3 μL of stop solution, and then the samples were dialyzed to remove free biotin. Then, protein concentration was measured by the Trp method and 3.3 μg of indicated biotinylated samples were incubated overnight at 4 °C with gentle shaking on each subarray on 400 μL of blocking buffer. After washing, bound proteins were detected with the incubation of Cy3‐streptavidin during 2 h at room temperature. Finally, after washing, the slides were dried by centrifugation at 260 *g* for 10 min and scanned at 532 nm. Then, the slides were scanned on the GenePix 4000B (Axon, Scottsdale, AZ, USA) and the images were generated and processed with the genepix pro 7.1 scanarray software [11, 12].

Analysis, normalization, and quantification of all microarray images were performed using the genepix pro 7.1 software as previously reported [10, 11, 12, 13, 14]. Ratios ≥ 1.5 or ≤ 0.67 of HCT116‐∆Np73 versus Mock control cells were used as cut‐off to determine protein expression alterations, as previously done [15, 16].

#### 2.2.2 SILAC cell culture and sample preparation for SILAC analyses

For metabolic labeling, HCT116‐∆Np73 or control cells were grown and maintained in DMEM containing either light [^12^C_6_]‐l‐lysine and [^12^C_6_]‐l‐arginine or heavy [^13^C_6_]‐l‐lysine and [^13^C_6_]‐l‐arginine (Dundee Cell Products, Dundee, Scotland, UK) supplemented with 10% dialyzed FBS, 100 units·mL^−1^ of penicillin/streptomycin at 37 °C in 5% CO_2_. At least eight duplications were performed to achieve > 98% incorporation of the heavy amino acids, which was determined as previously described [7]. We carried out forward and reverse experiments to avoid labeling bias in the study.

Preparation of conditioned medium was performed as indicated above, incubating cells for 48 h in serum‐free DMEM supplemented with heavy or light amino acids. Cell viability, determined with 0.4% trypan blue solution (Invitrogen), was higher than 95%. Proteins in the conditioned medium were precipitated with methanol/chloroform. Protein content was quantified by fluorescence using the Trp method, and subsequently 25 μg of protein from HCT116‐∆Np73 or control cells SILAC conditioned medium were mixed at a 1 : 1 ratio and run at 25 mA per gel in 15% SDS/PAGE. Gels were stained with the Colloidal Blue staining kit (Invitrogen), and lanes containing forward and reverse labeling experiments were cut into 10 slices. Excised bands were cut into small pieces and destained with 50 mm ammonium bicarbonate, 50% Acetonitrile (ACN), dehydrated with ACN, and dried. Gel pieces were rehydrated with 12.5 ng·μL^−1^ trypsin in 50 mm ammonium bicarbonate and incubated overnight at 30 °C. Peptides were extracted at 37 °C using 100% ACN and then 0.5% TFA, dried, cleaned using ZipTip with 0.6 μL C18 resin (Millipore, Burlington, MA, USA), and reconstituted in 5 μL of 0.1% formic acid, 2% ACN, prior to mass spectrometry (MS) analysis, which was performed as previously described [7].

#### 2.2.3 Mass spectrometry analysis, protein identification and SILAC quantification

Peptides were trapped onto a C18‐A1 ASY‐Column 2 cm precolumn (Thermo‐Scientific, Waltham, MA, USA), and then eluted onto a Biosphere C18 column (C18, inner diameter 75 μm, 10 cm long, 3 μm particle size) (NanoSeparations, Nieuwkoop, the Netherlands) and separated using a 170 min gradient from 0% to 35% Buffer B (Buffer A: 0.1% formic acid/2% ACN; Buffer B: 0.1% formic acid in ACN) at a flow‐rate of 300 nL·min^−1^ on a nanoEasy HPLC (Proxeon, Odense, Denmark) coupled to a nanoelectrospay ion source (Proxeon). Mass spectra were acquired on an LTQ‐OrbitrapVelos mass spectrometer (Thermo‐Scientific) in the positive ion mode. Full‐scan MS spectra (*m/z* 400–1200) were acquired in the Orbitrap with a target value of 1 000 000 at a resolution of 60 000 at *m/z* 400 and the 15 most intense ions were selected for collision induced dissociation fragmentation in the linear ion trap with a target value of 10 000 and normalized collision energy of 35%. Precursor ion charge state screening and monoisotopic precursor selection were enabled. Singly charged ions and unassigned charge states were rejected. Dynamic exclusion was enabled with a repeat count of 1 and exclusion duration of 30 s. Mass spectra *.raw files were searched against the Human Swiss Prot database (SwissProt_57.15.fasta) using mascot search engine (version 2.3; Matrix Science, London, UK) through proteome discoverer (version 1.4.1.14; Thermo, Waltham, MA, USA). Search parameters included a maximum of two missed cleavages allowed, carbamidomethylation of cysteines as a fixed modification and oxidation of methionine, N‐terminal acetylation and ^13^C‐Arg, and^13^C‐Lys as variable modifications. Precursor and fragment mass tolerance were set to 10 p.p.m. and 0.8 Da, respectively. Identified peptides were validated using Percolator algorithm with a *q*‐value threshold of 0.01. Relative quantification of identified peptides was performed using proteome discoverer. For each SILAC pair, proteome discoverer determines the area of the extracted ion chromatogram and computes the ‘heavy/light’ ratio. Protein ratios are then calculated as the median of all the unique quantified peptides belonging to a certain protein. The ratios among proteins in their heavy and light versions were used as fold‐change. The fold change cut‐off for dysregulated proteins was calculated using a permutation‐based test as described [8]. Proteins were quantified with at least one peptide hit in forward and reverse experiments and a variability ≤ 20%. A multipoint normalization strategy was applied to normalize the data sets against the 5% trimmed mean values, which is a robust statistical measure of central tendency that normalize most of the protein ratios to 1. Briefly, the 5% of the most extreme outliers – values – were removed and the mean of the 95% remaining data was determined, and used to normalize the ratio values, and thus, minimizing the effect of these extreme outliers and centering the log_2_ ratio distribution to zero. Since metabolic conversion arginine/proline can affect quantification accuracy in some cell types, we investigated arginine to proline conversion in HCT116 cells. Using heavy proline as a variable modification, less than 1% of proline‐containing peptides were heavy labeled in HCT116 cells.

### 2.3 Bioinformatics and statistical analysis

DAVID Database 6.8 was used to investigate for the enrichment of subcellular fractions, functional activity and deregulated functions of the quantified proteins of our proteomic dataset. STRING (http://string‐db.org/) was used to predict biological functions and identify clusters of interacting proteins [9, 17, 18]. STRING version 10.5 and MCL clustering enrichment 2 with the default 0.4 confidence score were used to identify the interacting partners in the dataset. Exocarta database was used to identify differentially released proteins previously identified in exosomes and to correlate them with externalization through non‐classical secretion [19].

### 2.4 WB and dot‐blot analysis

Protein extracts from conditioned medium or protein extracts from CRC cells were prepared, and used in WB and dot‐blot analyses as previously reported [7, 14]. For WB analyses, alternatively 25 μg of each CRC cell protein extract or tissue protein extract, or 5 μg of each conditioned medium were run in parallel in 10% SDS/PAGE. Then, proteins were transferred to nitrocellulose membranes (Hybond‐C extra; GE‐Healthcare, Chicago, IL, USA) using wet transfer (Bio‐Rad, Hercules, CA, USA). Alternatively, for dot‐blot analysis 10 μL of the secretome of HCT116 cells were deposited on nitrocellulose membranes. After blocking, membranes were incubated with specific mono‐ or polyclonal antibodies against the indicated proteins. Membranes were incubated at optimized dilutions with primary antibodies followed by incubation with either HRP‐anti‐mouse IgG (Sigma, St. Louis, MO, USA) at 1 : 2500 dilution, HRP‐anti‐rabbit IgG (Sigma) at 1 : 2500 dilution, or HRP‐streptavidin 1 : 1000 (RayBiotech, Norcross, GA, USA). Specific reactive proteins were visualized with SuperSignal West Pico Maximum Sensitivity Substrate (Pierce, Waltham, MA, USA). A total of five different antibodies at optimized dilutions were used (Table S1). Protein bands were quantified by densitometry using quantity one program (BioRad). All quantitations were normalized using actin or Red Ponceau staining as internal controls.

### 2.5 RNA extraction, semi‐quantitative PCR and qPCR

RNA was extracted from cell lines with the RNeasy Mini Kit (Qiagen, Limburg, the Netherlands) and quantified with a NanoDrop ND‐1000 spectrophotometer (Thermo Fisher Scientific). cDNA was synthesized using the Superscript III First Strand Synthesis kit (Invitrogen).

For semiquantitative reverse transcriptase‐PCR (RT‐PCR), reactions were performed using specific primers for each of the 10 analyzed genes (Table S1), in triplicate. PCR products were separated on 2% agarose gel, stained with GelRed (Biotium, Hayward, CA, USA), and quantified by densitometry using quantity one program (BioRad). All quantitations were normalized using GAPDH as internal control.

Quantitative real‐time PCR was performed in a Light Cycler apparatus (Roche Diagnostics, Basel, Switzerland) using the LightCycler‐FastStart DNA Master SYBR Green I Kit (Roche Diagnostics). Each reaction was performed in a final volume of 20 μL containing 2 μL of the cDNA product sample, 0.5 μmol·L^−1^ of each primer, and 1× reaction mix including FastStar DNA polymerase, reaction buffer, deoxyribonucleotide triphosphates, and SYBR green. Primer sets for ΔNp73, EMAP‐II, BDNF, p21 and p16 (Table S1) and the reaction conditions were as described previously [20, 21, 22]. The housekeeping gene succinate dehydrogenase complex subunit A (SDHA) was used to normalize gene expression results.

### 2.6 Plasma samples and ELISAs

Plasma samples with indicated pathological conditions were obtained from the IdISSC Biobank of the Hospital Clínico San Carlos (Madrid, Spain). A total of 60 plasma samples from CRC patients, premalignant colorectal individuals (low‐ and high‐grade colorectal adenomas), and control individuals were used in the study (Table S2). Written informed consent was obtained from all patients.

The Institutional Ethical Review Board of the IdISSC, Hospital Clínico San Carlos, ISCIII, and the Autonomous University of Madrid approved this proteomic analysis of ∆Np73 and its validation as biomarkers of CRC. The ethical aspects and procedures laid down in the Declaration of Helsinki.

ELISA kits were purchased from RayBiotech (VEGF‐C, VEGFR‐3, and BDNF), and Elabscience (EMAP‐II). Specificity of the ELISA kits was verified by the manufacturers. ELISA experiments were carried out using the recommended dilution for conditioned medium or plasma analyses in each case and according to the instructions of the manufacturer. The sensitivity of the ELISA kits for detection of VEGF‐C, VEGFR‐3, BDNF, and EMAP‐II was 15, 15, 80, and 75 pg·mL^−1^, respectively.

For the analysis of ELISA data sets, a one‐tailed Student's *t*‐test was performed assuming unequal variances to assess whether the means of control individuals and CRC groups were statistically different from each other. *P* values < 0.05 were considered statistically significant. Each individual protein was evaluated as marker in plasma of CRC patients and control individuals by a receiver operating characteristic (ROC) curve. All statistical analyses were done with Microsoft Office Excel. ROC curves were constructed with the r program (version 3.2.3), and the corresponding area under the curve (AUC) and the maximized sensitivity and specificity values were calculated using the r packages ModelGood and Epi.

### 2.7 Functional cell‐based assays

#### 2.7.1 Proliferation assay

For the proliferation assays 2 × 10^4^ cells were seeded in quadruplicates in 96‐well E‐plates to carry out an MTT cell proliferation assay (Cayman Chemical Company, Ann Arbor, MI, USA). At 24, 48, and 72 h post‐transfection, MTT reagent was added and absorbance was measured on a microplate reader at 570 nm (Multiskan Ex; Thermo Scientific, Waltham, MA, USA).

#### 2.7.2 Migration assay

For migration assays HUVECs or HLECs (40 000 cells per well) were seeded in the top of 24‐well cell culture inserts (Falcon®; #353097) incorporating polyethylene terephthalate with 8 μm pores. Previously, cells were treated with cell tracker™ Green CMFDA (Invitrogen; #C2925) following the manufacturer's indications. Conditioned media from HCT116‐ΔNp73 and Mock control cells were added in the lower well of a Falcon 24well companion plate. After 24–48 h post‐seeding, cells in the lower media and well were collected individually and washed with PBS 1× to eliminate media residues. Migrated cells were resuspended in 100 μL of PBS 1× and placed in a Costar 96 well black opaque plate. Then, fluorescence from the cell tracker was measured in a fluorimeter. In the case of the invasion assay, 24‐well cell culture inserts were pre‐coated with 500 μg·mL^−1^ of Matrigel GFR at 37 °C overnight.

#### 2.7.3 Transfection of BDNF and AIMP1 small interfering RNA

HCT116‐ΔNp73 stably transfected cells were cultured in ECM and transiently transfected with a Negative Control small interfering RNA (siRNA; Invitrogen™ *Silencer*™ Negative Control No1 siRNA AM4611), BDNF siRNA (Dharmacon™ ON‐TARGET Plus siRNA SMARTPool #L017626‐00‐0005, Lafayette, CO, USA) or AIMP1 siRNA (Sigma‐Aldrich MISSION^R^ esiRNA EHU075101, St. Luois, MO, USA) using JetPrime (Polyplus Transfection, Illkirch, France) according to the manufacturer's recommendations. In the BDNF siRNA transfection, at 24 h post‐transfection, medium's cells were collected individually and BDNF mRNA was measured. In the AIMP1 siRNA transfection, at 48 post‐transfection, medium's cells were collected individually and AIMP1 mRNA was measured. After optimization of transfection times, the interference of mRNAs was observed the better at 24 h for BDNF and 48 h for AIMP1.

#### 2.7.4 Tube formation assay

HCT116‐ΔNp73 and mock cells were seeded at 6 × 10^5^ cells on 100 mm dishes with DMEM. At 6–8 h post‐seeding, DMEM was replaced for 10 mL of ECM for each 100 mm dish. Conditioned media were collected after 72 h without reaching maximum confluence. Conditioned media were centrifuged at 405 *g* for 6 min to precipitate cells and recollect the cell‐free media.

Matrigel (growth factor reduced) (BD Bioscience, East Rutherford, NJ, USA) was placed in μ‐Slide angiogenesis (Ibidi; #81506, Gräfelfing, Germany) plates following the manufacturer's recommendations. HLEC or HUVEC cells were seeded in the wells with conditioned media from HCT116‐ΔNp73or Mock control cells and BDNF or AIMP1 siRNA transfection. Previously, conditioned media were refreshed with 25% of ECM medium. At the endpoint of each assay, images were acquired using a Cell Observer microscope. The formation of tube‐like structures was quantified using the Angiogenesis analyzer (imagej, NHI, Bethesda, MA, USA).

#### 2.7.5 Wound healing assay

Cells were grown on six‐well dishes. Upon confluency cells were scratched using a 200‐μL tip. Wound scratch was photographed under an inverted microscope every 2 h. Scratch area was calculated using the imagej software. Wound healing rate was calculated as the width at 0 h minus wound width at 60 h and expressed as speed of closure per hour [23]. Experiments were performed five times.

### 2.8 *In vivo* experiments in immunosuppressed mice

A group of 10 female nu/nu mice (Charles River, Wilmington, MA, USA) aged 6 weeks per group, were inoculated with stably transfected ΔNp73 or Mock control HCT116 cells. After 3 weeks mice were sacrificed and lung, kidney and livers were resected and evaluated for macrometastasis. All procedures were approved by the Institutional Organism for Animal Welfare (OEBA) and according to the European, Spanish, and local regulations.

## 3 Results

We have here performed the analysis of the secretome of HCT116‐∆Np73 CRC cells and control cells using antibody microarrays‐driven proteomics for the profiling of alterations in 493 immunomodulators, cytokines, chemokines, adipocytokines, growth factors, proteases, and other secreted proteins, which are usually missed in mass‐spectrometry quantitative experiments. Then, to get the most comprehensive analysis of altered secreted proteins associated to the overexpression of ∆Np73, a quantitative SILAC analysis was also performed. A schematic representation of the work‐flow of the study, from collection of the secretome of CRC cells, screening of the antibody microarrays and SILAC data analysis and bioinformatics, to validation of the protein alterations by PCR, WB, dot‐blot, ELISA, and functional experiments is depicted in Fig. S1.

### 3.1 Profiling of ∆Np73‐associated protein alterations in the secretome of HCT116 CRC cells by antibody microarrays

**Fig. 1:**
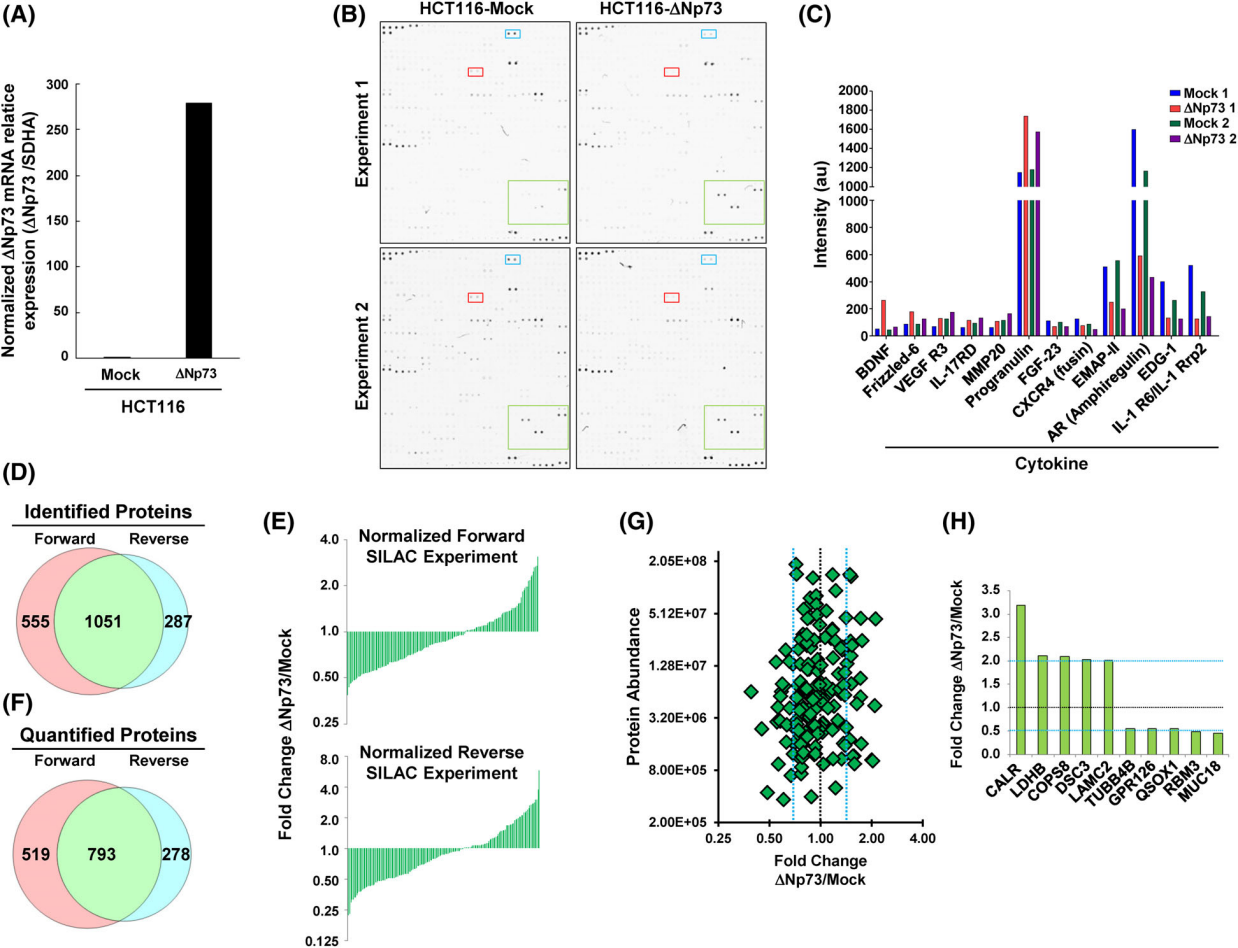
The overexpression of ∆Np73 in CRC cells produced the dysregulation of cytokines, growth factors, cell signaling proteins, and proteins related to tumorigenesis, metastasis and cell–cell adhesion as determined by antibody microarrays and SILAC quantitative proteomic analyses. (A) Confirmation by qPCR of the overexpression of ∆Np73 in stably transfected HCT116 CRC cells in comparison to mock control cells. Triplicates were analyzed (*n* = 3). (B) The secretome of two different biological replicates of HCT116‐∆Np73 CRC cells and mock control cells was analyzed using L‐series antibody microarrays. (C) Microarrays were incubated with biotin‐labeled samples followed with Cy3‐streptavidin, and differential expression for proteins due to ∆Np73 overexpression was found in both biological replicates. a.u., arbitrary units. (D) For the SILAC approach, protein extracts from the conditioned media from metabolically labeled HCT116‐∆Np73 and mock cells were mixed 1 : 1 to perform forward and reverse experiments and run on SDS‐polyacrylamide gels. A total of 8730 and 6862 peptides in forward and reverse experiments were found, resulting in 1586 and 1338 identified proteins, respectively, with 1051 proteins in common in both experiments. (E, F) After data normalization using the 5% trimmed mean (E), 1590 proteins were quantified in both SILAC experiments (F), with 793 proteins quantified in common. (G) A total of 37 dysregulated proteins by ∆Np73 identified in the secretome of HCT116 cells with variability ≤ 20%, and fold‐change ≥ 1.5. At least two peptides in both biological replicates, with 19 and 18 up‐ and down‐regulated proteins, respectively, were observed. (H) The top 10 dysregulated proteins observed by SILAC were represented by a bar‐graph.

First, we confirmed the overexpression of ∆Np73 stably transfected HCT116 CRC cells in comparison to control cells by qPCR (Fig. 1A).

We then investigated the secretome of two different biological replicates of HCT116‐∆Np73 CRC cells and control cells for alterations in the expression of immunomodulators, cytokines, chemokines, adipocytokines, growth factors, and proteases using the L‐series antibody microarrays. Microarray images of the indicated protein samples labeled with biotin followed by incubation with Cy3‐streptavidin are shown in Fig. 1B.

From the 493 chemokines, proangiogenic, and growth factors analyzed, we observed a differential expression in both biological replicates for 14 of them (Fig. 1C; Table 1), with BDNF, Frizzled‐6 and VEGF R3 as the most upregulated and IL‐1 RRP2, EDG‐1, Amphiregulin and EMAP‐II as the most downregulated factors by ∆Np73 overexpression.

**Table 1:** Proteins differentially regulated by ∆Np73 identified by antibody microarrays and SILAC in the secretome of HCT116 CRC cells with at least 1.5‐fold change variation.

	Name	Description	Uniprot accession	Mean fold change Δnp73/mock	ΣCoverage	Σ# unique peptides	Σ# peptides	Σ# PSMs	DAVID^a^	Exocarta
Antibody microarray	BDNF	Brain‐derived neurotrophic factor	P23560	3.19	–	–	–	–		Y
	FZD6	Frizzled‐6	O60353	1.72	–	–	–	–		Y
	FLT4 (VEGFR3)	Vascular endothelial growth factor receptor 3	P35916	1.64	–	–	–	–		N
	IL17RD	Interleukin‐17 receptor D	Q8NFM7	1.64	–	–	–	–		N
	MMP‐20	Matrix metalloproteinase‐20	O60882	1.56	–	–	–	–	Extracellular space	N
	IL20	Interleukin‐20	Q9NYY1	1.46	–	–	–	–	Extracellular space	N
	GRN	Progranulin	P28799	1.42	–	–	–	–	Both	Y
	LCN1	Lipocalin‐1	P31025	1.41	–	–	–	–	Both	Y
	FGF23	Fibroblast growth factor 23	Q9GZV9	0.66	–	–	–	–	Extracellular space	N
	CXCR4	C‐X‐C chemokine receptor type 4	P61073	0.59	–	–	–	–	Exosome	Y
	EMAP‐II (AIMP1)	Aminoacyl tRNA synthase complex‐interacting multifunctional protein 1	Q12904	0.43	–	–	–	–	Extracellular space	Y
	AR	Amphiregulin	P15514	0.37	–	–	–	–	Extracellular space	Y
	S1PR1 (EDG‐1)	Sphingosine 1‐phosphate receptor 1	P21453	0.41	–	–	–	–		N
	IL‐36R (IL‐1Rrp2)	Interleukin‐1 receptor‐like 2	Q9HB29	0.34	–	–	–	–		N
Secretome SILAC	Calreticulin variant	Calreticulin variant	Q53G71	3.19	20.44	6	6	21		Y
	LDHB	l‐Lactate dehydrogenase B chain	P07195	2.11	39.82	10	11	133	Exosome	Y
	COPS8	Isoform 2 of COP9 signalosome complex subunit 8	Q99627	2.09	39.38	4	4	17	Exosome	Y
	DSC3	Isoform 3B of desmocollin‐3	Q14574	2.03	3.22	3	3	46		Y
	LAMC2	Isoform short of laminin subunit gamma‐2	Q13753	2	7.74	7	7	13	Extracellular space	Y
	GLO1	Isoform 2 of lactoylglutathione lyase	Q04760	1.76	34.32	6	6	31	Exosome	Y
	APEX1	DNA‐(apurinic or apyrimidinic site) lyase	G3V5Q1	1.74	23.97	5	5	11		Y
	MSN	Moesin	P26038	1.72	28.25	10	18	84	Both	Y
	SRI	Sorcin	C9J0K6	1.72	14.84	2	2	7	Exosome	Y
	DDB1	DNA damage‐binding protein 1	Q16531	1.72	16.14	16	16	41	Both	N
	PYGL	Isoform 2 of Glycogen phosphorylase, liver form	P06737	1.64	22.51	14	17	49	Exosome	Y
	PFAS	Phosphoribosylformylglycinamidine synthase	A8K8N7	1.58	3.29	3	3	5	Exosome	Y
	MARCKSL1	MARCKS‐related protein	P49006	1.57	14.36	2	2	13	Exosome	Y
	Aspartate aminotransferase	Aspartate aminotransferase	B3KUZ8	1.55	13.75	4	4	9		N
	DSG2	Desmoglein‐2	Q14126	1.53	16.82	13	13	56	Exosome	Y
	ATP5F1	ATP synthase F(0) complex subunit B1, mitochondrial	Q5QNZ2	1.52	35.9	6	6	18	Exosome	N
	LGALS3BP	Galectin‐3‐binding protein	Q08380	1.51	33.16	15	15	117	Both	Y
	TIMP2	TIMP metallopeptidase inhibitor 2	24R8V7	1.51	26.26	5	5	21		Y
	VDAC1	Voltage‐dependent anion‐selective channel protein 1	P21796	1.5	49.47	9	10	33	Exosome	Y
	EIF3I	Eukaryotic translation initiation factor 3 subunit I	Q13347	0.67	20	6	6	21	Exosome	Y
	CDH3	Isoform 2 of Cadherin‐3	P22223	0.66	12.63	6	6	29		Y
	CAPZB	Capping protein (actin filament) muscle Z‐line, beta	B1AK87	0.66	38.08	4	10	29	Exosome	Y
	PDCD6IP	Programmed cell death 6‐interacting protein	Q8WUM4	0.63	20.51	13	13	22	Exosome	Y
	PTPRF	Isoform 2 of Receptor‐type tyrosine‐protein phosphatase F	P10586	0.63	9.22	13	13	26	Exosome	Y
	LGALS1	Galectin‐1	P09382	0.62	42.22	5	5	87	Both	Y
	CRABP2	Cellular retinoic acid‐binding protein 2	P29373	0.61	61.59	7	7	35	Exosome	Y
	LRRFIP1	Isoform 3 of Leucine‐rich repeat flightless‐interacting protein 1	Q32MZ4	0.61	5.05	3	3	7		Y
	COL6A1	Collagen alpha‐1(VI) chain	87X0S5	0.59	16.28	12	12	34		Y
	PVR	Poliovirus receptor	B3KSS4	0.59	6.91	2	2	18		Y
	WDR1	WD repeat‐containing protein 1	O75083	0.58	21.12	10	10	38	Exosome	Y
	TLN1	Talin‐1	Q9Y490	0.58	9.88	16	16	39	Exosome	Y
	RPA1	Replication protein A 70 kDa DNA‐binding subunit	I3L4R8	0.56	21.41	5	5	11		Y
	TUBB4B	Tubulin beta‐4B chain	P68371	0.56	35.73	4	11	43	Exosome	Y
	GPR126	Isoform 2 of G‐protein coupled receptor 126	Q86SQ4	0.55	3.6	3	3	11		N
	QSOX1	Sulfhydryl oxidase 1	O00391	0.55	19.68	12	12	37	Both	Y
	RBM3	RNA‐binding protein 3	P98179	0.49	27.39	3	3	8		Y
	MUC18	Cell surface glycoprotein MUC18	B3KXZ9	0.45	16.95	6	6	13		N

^a^ Presence in extracellular space, in exosomes, or in both locations as retrieved from Gene Ontology from DAVID is indicated.

### 3.2 Identification and quantification of protein alterations in conditioned media using SILAC

HCT116‐∆Np73 and mock cells were metabolically labeled in SILAC medium at least for eight doublings to perform forward and reverse experiments quantitative proteomic analyses.

Conditioned media from metabolically labeled cells were harvested after 48 h, concentrated, and quantified. Protein extracts from the conditioned media were mixed 1 : 1 to perform forward and reverse SILAC experiments and run on SDS‐polyacrylamide gels. Then, 10 bands were excised and in‐gel digested with trypsin. Peptides were separated using reverse phase and analyzed on a linear ion trap OrbitrapVelos mass spectrometer (Thermo Scientific). A total of 8730 and 6862 peptides were identified in forward and reverse experiments resulting in 1586 and 1338 identified proteins, respectively, with 1051 proteins in common in both experiments (Fig. 1D; Table S3). For quantification, peptide ratios were calculated using proteome discoverer by comparing the intensities of the light‐ and heavy‐labeled precursor ions at high resolution. Proteins were quantified with at least one peptide hit in forward and reverse experiments. ∆Np73/Mock ratios among proteins in the heavy and light versions were used as fold‐change. We normalized the data sets against the 5% trimmed mean to minimize the effect of extreme outliers and to center the protein fold change distribution to one (Fig. 1E) [24]. In total, 1590 proteins were quantified in both SILAC experiments, with 793 proteins quantified in common (Fig. 1F).

After normalization, by using a permutation‐based statistical test, we fixed a fold‐change of ≥ 1.5 (mean of two experiments) as significant for identification of dysregulated proteins [7, 15]. In total, we found 37 dysregulated proteins by ∆Np73 in the secretome of HCT116 cells with variability ≤ 20%, fold‐change ≥ 1.5, and at least two peptides per protein in each biological replicate. Among them, 19 and 18 proteins showed up‐ and down‐regulation, respectively (Fig. 1G, Table 1). From the total of quantified proteins, we observed with about a twofold change dysregulation CALR, LDHB, COPS8, DSC3, and LAMC2 as the top up‐regulated proteins, and TUBB4B, GPR126, QSOX1, RBM3, and MUC18 as the most downregulated factors by ∆Np73 overexpression (Fig. 1H).

According to Gene Ontology analysis, among the top five enriched fractions, extracellular exosome and proteins from the extracellular space represented the 64.7% of the total number of quantified dysregulated proteins (33 proteins out of 51 dysregulated proteins in total, Table 1). In addition, 26 proteins with localization on cytoplasm or cytosol and 16 plasma membrane proteins were observed. Moreover, to analyze if quantified proteins could be present in exosomes, we used the Exocarta database, which contains proteins identified in exosomes via proteomic analyses [19]. Thirty‐nine of 51 proteins had been previously identified in exosomes (Table 1). Therefore, in total, from both databases 43 of 51 dysregulated proteins (84.3%) have been previously identified either in exosomes or in the extracellular space, and thus, validating the presence of the proteins of the dataset in the secretome.

### 3.3 Biological function, pathway analysis, and interaction networks for dysregulated proteins induced by ∆Np73

In total, from antibody microarrays and SILAC proteomics analyses, 51 proteins were found to be dysregulated by ∆Np73 in the HCT116 cells, with 27 and 24 proteins showing up‐regulation and down‐regulation (Table 1).

Among them, it was observed that the dysregulation of seven receptors (FLT4 ‐VEGFR3‐, IL17RD, PTPRF, CXCR4, S1PR1, IL‐36R, and PVR) and two proteins (APEX1 and CRABP2) described as transcription factors, not previously described to be altered by ∆Np73. We also found several proteins (APEX1, GLO1, CDH3, PTPRF, QSOX1, DSG2, or DSC3) whose dysregulation has been associated to the tumorigenesis process, prognosis of patients and resistance to treatment [25, 26, 27, 28, 29]. In addition, as the most altered process modulated by ∆Np73, we found that about 20% of the dysregulated proteins were implicated in cell adhesion. Moesin, calreticulin, sorcin, DSG2, DSC3, and LGALS3BP were found to be upregulated and Talin‐1, CDH3, PTPRF, PDCD6IP, and COL6A1 downregulated by ∆Np73.

To identify altered biological functions that might play a role in CRC, differentially secreted proteins due to ∆Np73 were analyzed using STRING [30] (Fig. S2). Using MCL algorithm (value 2), we defined seven clusters containing three or more proteins. Among the three large clusters containing four interacting proteins, there was a cluster involved in cell adhesion and migration with CXCR4, S1PR1, LGALS1, and MCM, another cluster involving MSN, CALR, and CAPZB with the main edge on WDR1, which induces disassembly of actin filaments in conjunction with ADF/cofilin family proteins, and the last cluster containing APEX1, DDB1, RPA1, and COPS8 mainly involved in DNA binding and DNA repair binding.

In summary, ∆Np73 produces the dysregulation of cell adhesion proteins, transcription factors, receptors, and previously published proteins related to cancer or cancer metastasis, with most of them not previously identified as modulated by ∆Np73. All of them are producing important changes affecting cellular adhesion, cell growth, cell proliferation and cell death and survival, increasing the tumorigenesis and metastatic properties of CRC cells [31, 32].

### 3.4 Validation of dysregulated proteins

**Fig. 2:**
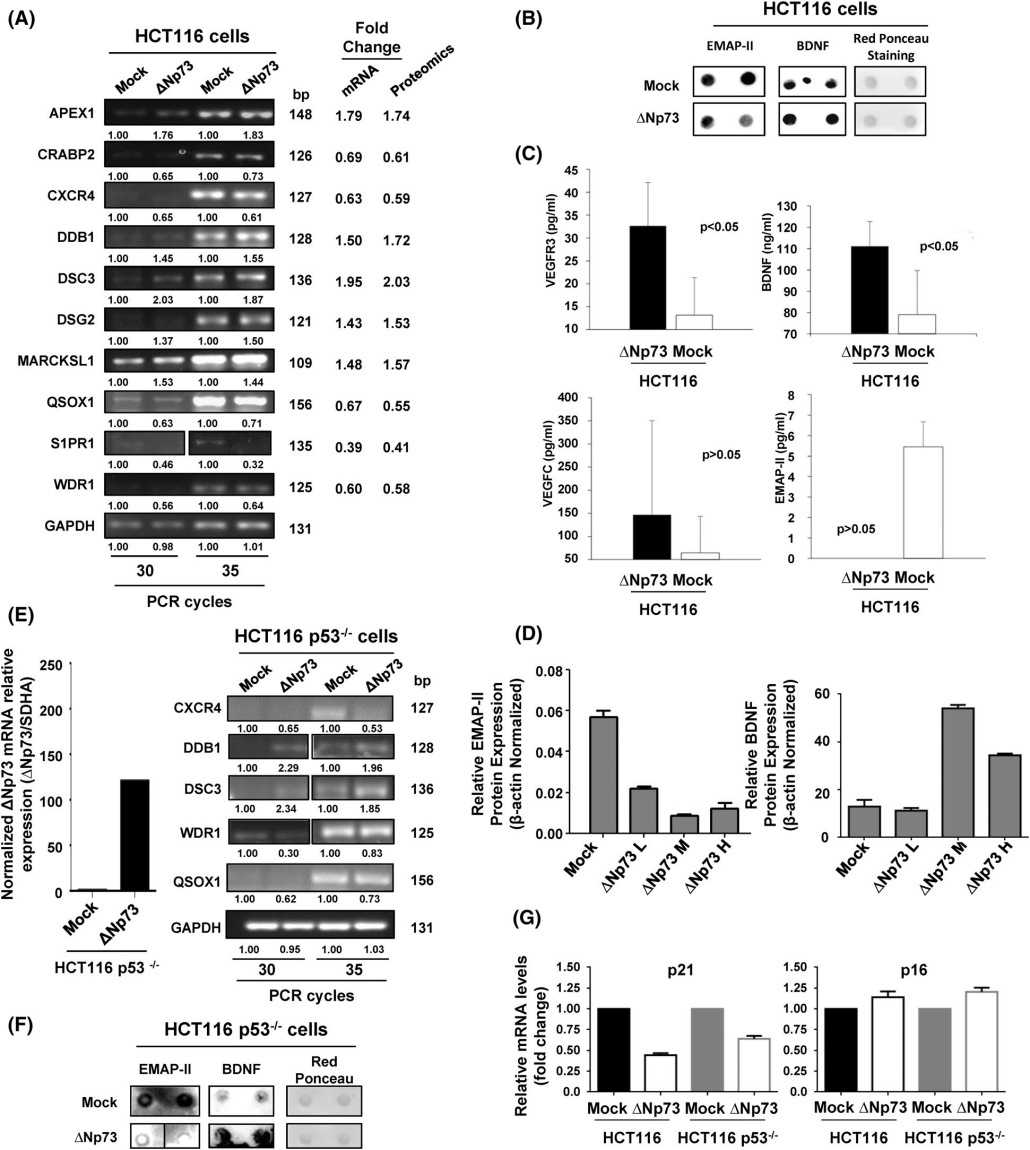
Validation of ∆Np73‐dysregulated proteins. Validation of the dysregulated proteins found was carried out using different methodologies. (A) Dysregulation of selected genes due to the stably overexpression of ∆Np73 was firstly confirmed by PCR in comparison to stably transfected mock control cells. The image is representative of four different experiments where 30 or 35 PCR cycles were performed. GAPDH was used as control of the assay. bp, base pair. (B) At protein level, the dysregulation of BDNF, and EMAP‐II was verified in the conditioned media of HCT116 mock control cells by dot‐blot. Red Ponceau staining was used as loading control. Two independent biological replicates were measured for each condition assessed. (C) Quantification of BDNF, VEGFC, and VEGFR3 in the conditioned medium or EMAP‐II in cell extracts was performed by ELISA. One‐tailed Student's *t*‐test was performed. All values are expressed as median ± standard deviation. Significant data were determined for VEGFR3 and BDNF, and almost significant for EMAP‐II (*P* = 0.06635). (D) Dysregulation of EMAP‐II and BDNF was also verified by WB, using β‐actin as loading control. Quantification data is shown. L, low; M, medium; H, high are referred to the ∆Np73 levels after cell sorting of HCT‐116 CRC cells, where a lower (EMAP‐II) or higher (BDNF) expression is observed according to the expression of ∆Np73. All results either at mRNA or protein level showed consistency with the alterations observed by proteomics. Triplicates were analyzed with Mann–Whitney *U*‐test. Error bars indicate standard deviation. (E) Left, confirmation by qPCR of the overexpression of ∆Np73 in stably transfected HCT116 p53^−/−^ CRC cells in comparison to mock control cells. Right, dysregulation of selected genes due to stably overexpression of ∆Np73 in HCT116 cells was confirmed by PCR in HCT116 p53^−/−^ cells in comparison to stably transfected mock control cells to demonstrate the presence of p53 was not affecting their dysregulation. The image is representative of four different experiments where 30 or 35 PCR cycles were performed. GAPDH was used as control of the assay. (F) The dysregulation of BDNF and EMAP‐II was also confirmed in the conditioned media of mock control and ∆Np73‐HCT116 p53^−/−^ cells by dot‐blot. Red Ponceau staining was used as loading control. Two independent biological replicates were measured for each condition assessed. (G) Dysregulation of the p53 target genes p21 and p16 in HCT116 and HCT116 p53^−/−^ ∆Np73 and mock control cells was analyzed by qPCR. Data were normalized with SDHA as reference gene. Triplicates were analyzed with Mann–Whitney *U*‐test. Error bars indicate standard deviation.

We performed an initial validation of ∆Np73‐dysregulated proteins by PCR, WB, dot‐blot and ELISA (Fig. 2). First, we tested by PCR those genes altered by the overexpression of ∆Np73 related to tumorigenesis as QSOX1, APEX1, MARCKSL1, CRABP2, CXCR4, or DDB1 and cell–cell adhesion as DSC3, DSG2, or WDR1. A good correlation was observed between the mRNA expression alteration by ∆Np73 and the fold‐change quantified by proteomics. Therefore, we confirmed the dysregulation of all analyzed mRNAs in stably transfected ∆Np73 and mock cells at similar extents than that previously observed by proteomics (Fig. 2A).

We next evaluated at protein level the dysregulation of BDNF, VEGFR3, EMAP‐II, and VEGFC by dot‐blot, ELISA and/or WB analyses (Fig. 2B–D). BDNF, VEGFR3, VEGFC, and EMAP‐II dysregulation was confirmed at protein level by dot‐blot and/or ELISA, with similar dysregulation observed between both stably transfected cells with that observed by proteomics (Fig. 2B,C). In addition, the protein dysregulation of EMAP‐II and BDNF by ∆Np73 overexpression was further evaluated by WB with specific antibodies and stably transfected HCT116 CRC cells with low, medium, and high levels of ∆Np73 as determined by qPCR after cell sorting (data not shown). The protein content of EMAP‐II and BDNF in HCT116 cells correlated with the ∆Np73 levels (Fig. 2D and Fig. S3).

Finally, to further confirm these proteins as effectors of ∆Np73 and that the presence of p53 was not‐affecting their dysregulation, the secretome and the total RNA of HCT116 p53^−/−^‐∆Np73 and mock control cells were analyzed. First, ∆Np73 overexpression was confirmed in HCT116 p53^−/−^‐∆Np73 in comparison to mock control cells. Then, selected markers were analyzed by PCR. All of them (CXCR4, DDB1, DSC3, WDR1, and QSOX1) showed dysregulation at similar extents in HCT116 and HCT116 p53^−/−^ cells upon ∆Np73 overexpression (Fig. 2E). Next, we analyzed the dysregulation of BDNF and EMAP‐II at protein level in the secretome of HCT116 p53^−/−^‐∆Np73 and mock cells by dot‐blot analysis (Fig. 2F). A good correlation was observed between their protein expression alteration by ∆Np73 in HCT116 p53^−/−^ and HCT116 cells (Fig. 2B,F). Finally, we analyzed the expression levels of the p53 target genes p21 and p16 in HCT116 and HCT116 p53^−/−^ cells upon ∆Np73 overexpression by qPCR (Fig. 2G). p21 and p16 showed similar expression levels upon ∆Np73 overexpression in HCT116 p53^−/−^ and HCT116 cells.

Collectively, our results confirmed that although the expression of some p53 target genes could be affected by ∆Np73 overexpression, p53 should not be affecting the dysregulation of most of the identified effectors of ∆Np73 identified here by proteomics. In this sense, dysregulation of CXCR4, DDB1, DSC3, WDR1, and QSOX1 at mRNA level, and BDNF and EMAP‐II at protein level were observed at similar extents in HCT116 and HCT116 p53^−/−^ cells, which confirmed them as ∆Np73effectors.

### 3.5 BDNF, VEGFR3, VEGFC, and EMAP‐II analysis as blood‐based candidate biomarkers for colorectal cancer diagnosis

**Fig. 3:**
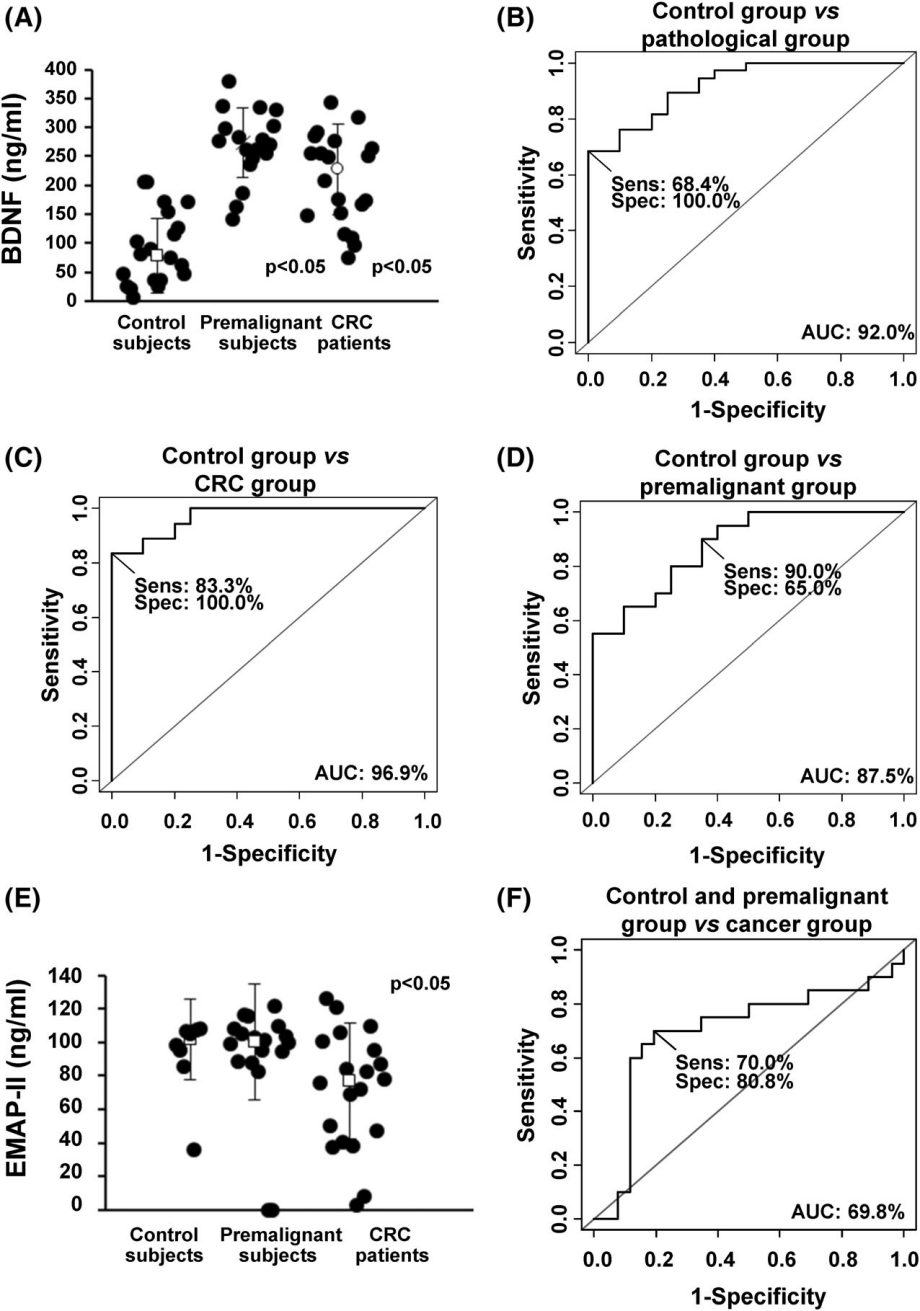
Evaluation of the plasma biomarker potential of selected identified proteins. (A) Quantification of BDNF using commercially available ELISAs in serum from control subjects, premalignant individuals, and CRC patients (*n* = 20 for each group). All values are expressed as median ± standard deviation. One‐tailed Student's *t*‐test was performed. (B–D) Determination of the BDNF value as discriminating plasma biomarker between control individuals and pathological subjects was carried out though ROC curves calculating their individual (B, C) and combined AUC (D). Sens, sensitivity; Spec, specificity. (E) Quantification of EMAP‐II using commercially available ELISAs in serum from control subjects (*n* = 8), premalignant individuals (*n* = 20), and CRC patients (*n* = 20). All values are expressed as median ± standard deviation. One‐tailed Student's *t*‐test was performed. (F) Determination of the EMAP‐II value as discriminating plasma biomarker between control + premalignant group and cancer group was carried out though ROC curves calculating AUC. BDNF and EMAP‐II plasma levels significantly discriminated between control and premalignant and CRC patients (*P* < 0.05).

Next, since there are evidences that ∆Np73 dysregulation in CRC takes place early during tumorigenesis [31], we hypothesized that some of the overexpressed effectors of ∆Np73 in the secretome could serve as plasma biomarkers of CRC. Thus, we tested and quantified the presence of BDNF, VEGFC, VEGFR3, and EMAP‐II effectors of ∆Np73 in plasma from CRC patients, patients with premalignant lesions and controls using commercially available ELISAs (Fig. 3; Fig. S3).

Using 60 plasma samples (20 from patients with premalignant cancer lesions, 20 CRC patients stage III and IV, and 20 from control individuals), levels in plasma for BDNF (median ± SD = 227.93 ± 78.75 ng·mL^−1^ for colorectal patients, 273.40 ± 60.99 ng·mL^−1^ for premalignant subjects, and78.05 ± 63.94 ng·mL^−1^ for controls), and EMAP‐II (101 665 ± 24 177 pg·mL^−1^ for controls, 100 590 ± 34 548 pg·mL^−1^ for premalignant subjects, and 76 985 ± 34 839 pg·mL^−1^ for CRC patients), significantly discriminated patients from controls samples for BDNF and EMAP‐II (*P*‐value < 0.05) (Fig. 3). We have also evaluated the correlation between the ΔNp73 mRNA levels in tissue and these proteins levels in plasma from patients with cancer (*n* = 6) and subjects with premalignant lesions (*n* = 17), obtaining a potential correlation for BDNF (*r* = 0.464 for patients with cancer and *r* = 0.552 for subjects with premalignant lesions) and for EMAP‐II (*r* = −0.813 for patients with cancer). On the other hand, values for VEGFC and VEGFR3 were not significant (Fig. S3).

In addition, we determined the usefulness as candidate biomarkers in plasma of the significant effectors able to discriminate CRC and premalignant individuals from controls (BDNF and VEGFR3) calculating its individual sensitivity and specificity by means of ROC curves. Individual AUC values for BDNF for discriminating premalignant individuals from controls were 96.9% (sensitivity 83.3% and specificity 100%), 87.5% (sensitivity 90% and specificity 65.0%) for CRC patients from controls, and 92% (sensitivity 68.4% and specificity 100%) for all patients from controls (Fig. 3B). Regarding EMAP‐II AUC value for discriminating controls and premalignant individuals from CRC patients was 69.8% (sensitivity 70% and specificity 80.8%) (Fig. 3F). For EMAP‐II analysis, 8 plasma samples from control subjects were available.

Collectively, these results confirm the predictive value of ∆Np73 effector BDNF as CRC biomarker in plasma of patients.

### 3.6 Implication of ΔNp73 and its effectors in metastasis formation, vasculogenesis and lymphangiogenesis

As many of the ∆Np73 putative effector targets identified in this study are related to the metastatic process, we decided to evaluate the homing and metastatic potential of HCT116 stably overexpressing ∆Np73 cells (HCT116‐∆Np73) and mock cells (HCT116‐mock) by tail vein injection. After 3 weeks post‐injection mice were sacrificed and lungs, liver and kidney were extracted and examined. Three out of 10 mice injected with HCT116‐∆Np73 presented lung metastasis versus none of the control mice, showing a statistical trend (*P* = 0.1; by the Fisher's exact test). In contrast, none control mice exhibited metastasis in any of the evaluated organs. A representative *ex vivo* lung metastasis is shown (Fig. S4). In concordance with the results observed in mice, those cells over‐expressing ∆Np73 promotes proliferation [33], and *in vitro* migration as a statistically significant wound healing rate was observed (Fig. S4).

**Fig. 4:**
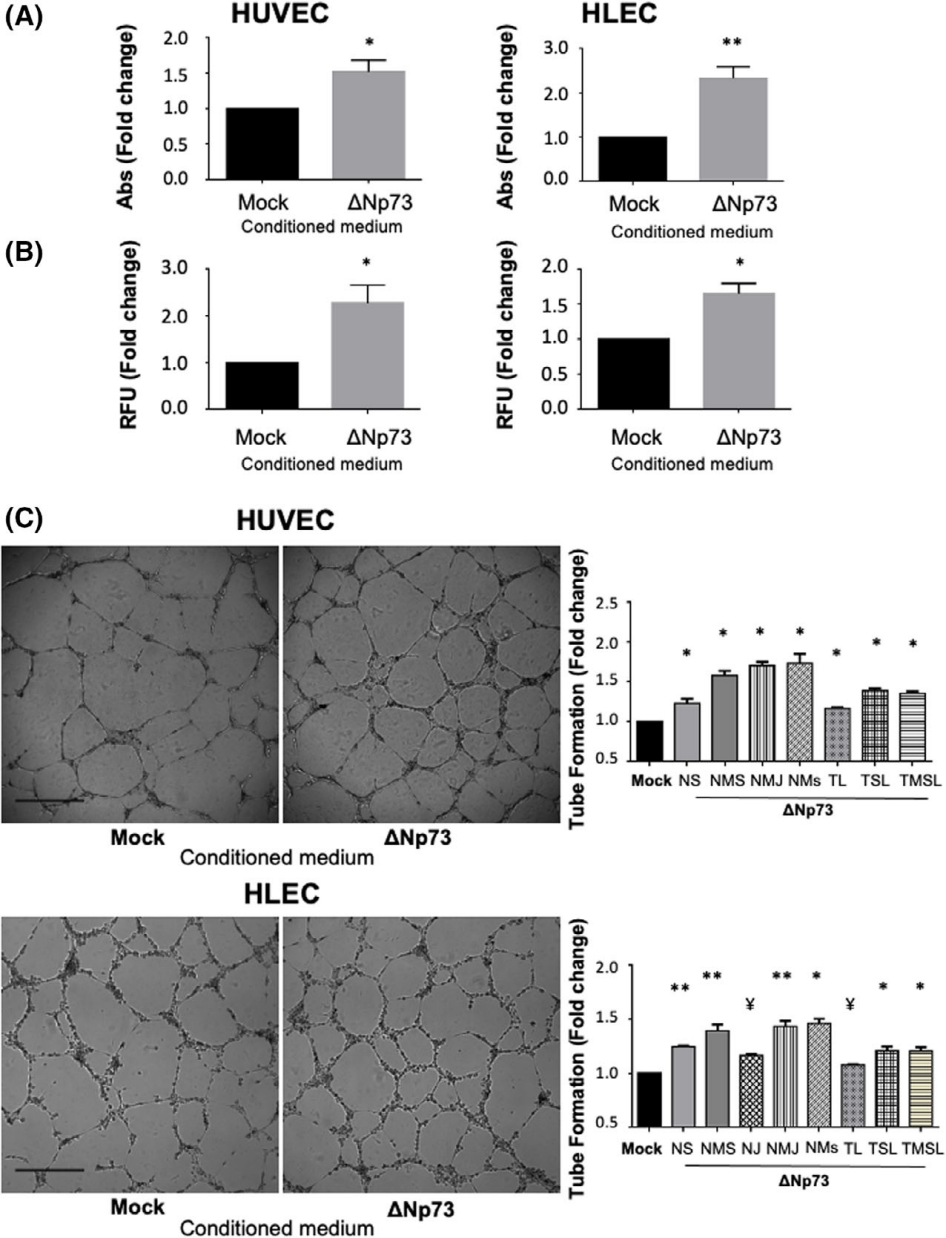
ΔNp73 promotes angiogenesis and lymphangiogenesis *in vitro*. (A–C) HUVEC and HLEC primary cells were treated with conditioned media from HCT116‐Mock and HCT116‐ΔNp73 colon cancer cells. (A) Proliferation potential performed with the MTT assay. Five replicates of each experimental condition were measured and analyzed with Mann–Whitney *U*‐test. Error bars indicate standard deviation. (B) Invasion capacity measured with a Transwell system using Matrigel as barrier. Five replicates of each experimental condition were measured and analyzed with Mann–Whitney *U*‐test. Error bars indicate standard deviation. (C) Cell tube formation (scale bar = 500 μm). The parameters recorded are NS (number of segments); NMS (number of master segments); NJ (number of junctions); NMJ (number of master junctions); NMs (number of meshes); TL (total length); TSL (total segment length); TMSL (total master segment length). Five replicates of each experimental condition were measured and analyzed with Mann–Whitney *U*‐test. Error bars indicate standard deviation. Statistical significance: *P < 0.05; **P < 0.01; ¥*P* = 0,06; RFU, fluorescence arbitrary units; Abs, absorbance.

**Fig. 5:**
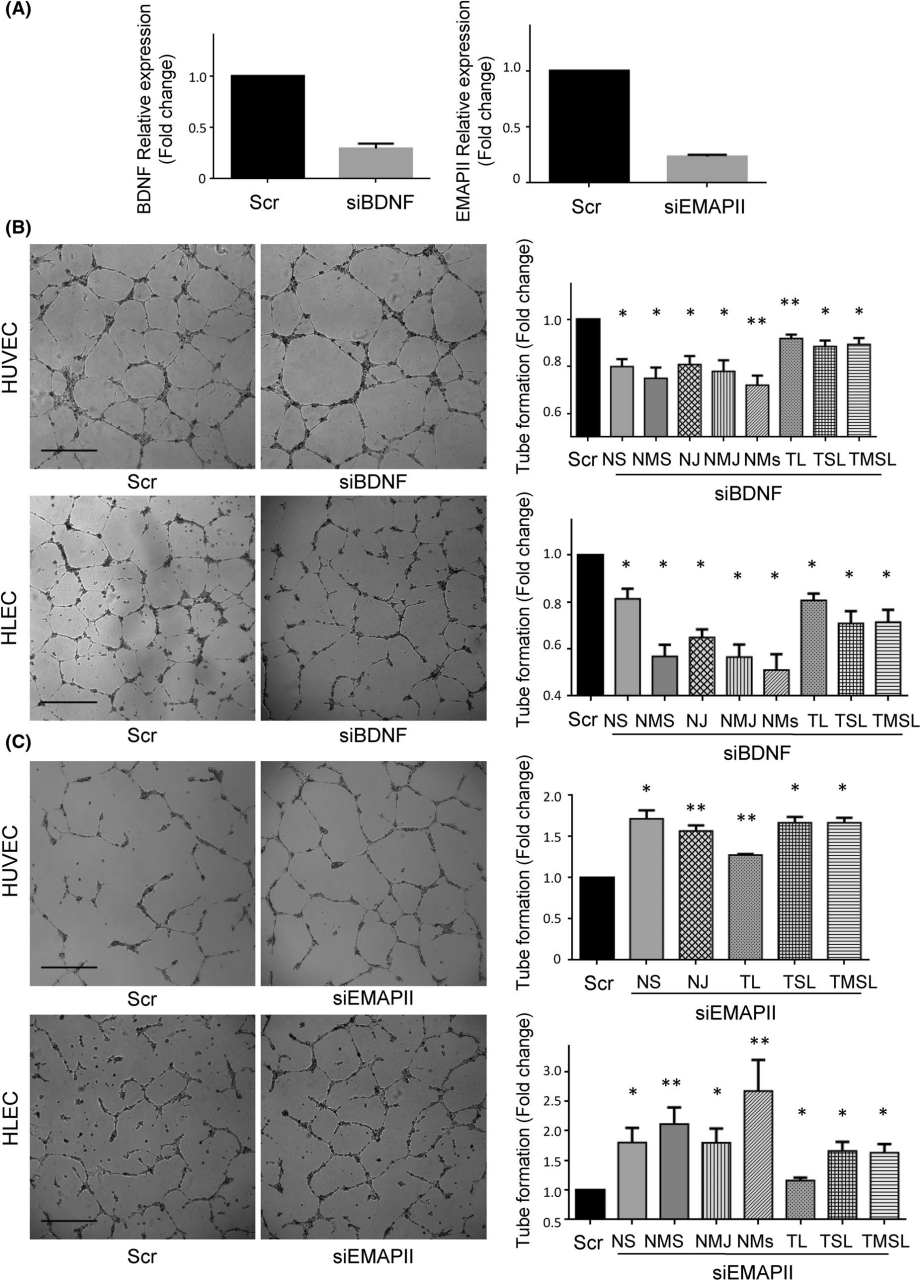
BDNF and EMAP‐II silencing effects on endothelial and lymphatic vasculature. (A) BDNF (left) and EMAP‐II (right) relative expression of HCT116‐ΔNp73 cells transiently transfected with a control siRNA (Scr) or a siRNA against BDNF (siBDNF) or EMAP‐II (siEMAP‐II). The efficiency of the siBDNF and siEMAP‐II was around 75% and 85%, respectively. (B, C) Tube formation assay (scale bar = 500 μm) in HUVEC and HLEC cells exposed to conditioned medium of HCT116‐∆Np73 treated with Scr or siBDNF or siEMAPII. The parameters recorded are NS (number of segments); NMS (number of master segments); NJ (number of junctions); NMJ (number of master junctions); NMs (number of meshes); TL (total length); TSL (total segment length); TMSL (total master segment length). Five replicates of each experimental condition were measured and analyzed with Mann–Whitney *U* test. Error bars indicate standard deviation. Statistical significance: *P < 0.05; **P < 0.01.

Since several of the top up‐ or down‐regulated proteins by ∆Np73 were linked to vasculogenesis and lymphangionesis, we carried out different cell‐based functional analysis to evaluate the implication of ∆Np73 and their effectors in these processes (Figs 4 and 5).

HCT116‐∆Np73 secretome clearly promotes proliferation and invasion of both HUVEC and HLEC primary cells compared to HCT116‐mock secretome (Fig. 4A,B). Images of the tube formation assay were quantitatively evaluated for number of segments, number of master segments, junctions, meshes, total length of vascular structures, total segment length, and total master length. Analysis revealed a statistically significant increase in these parameters in both cell types, indicating the paracrine implication of ∆Np73 secreted effectors in the formation of new blood and lymphatic vessels (Fig. 4C).

BDNF and EMAP‐II are known to play a role in angiogenesis and vascular patterning, thus, we sought to determine whether ∆Np73 promotion of vascular and lymphatic endothelial tube formation is supported through these factors. We performed siRNA silencing experiments of BDNF and EMAP‐II on HCT116 stably transfected cells with ∆Np73. The efficiency of siRNA down‐regulation on the targets was confirmed by PCR (Fig. 5A). Next, the conditioned medium of BDNF and EMAP‐II transiently silenced cells was used in the tube formation assays. A significant inhibition on blood and lymphatic vessel formation was observed with the silencing of BDNF (Fig. 5B), and as expected a significantly promotion of blood vasculature and lymphatic structures when interfering EMAP‐II (Fig. 5C).

## 4 Discussion

Cumulative data support ΔNp73 as the main TP73 isoform conferring oncogenic properties in CRC [31, 33, 34, 35, 36]. Therefore, since the mechanisms and target effectors underlying these oncogenic functions are currently of great interest, we decided to specifically study the target protein effectors of ΔNp73 in CRC, and functionally explored their role in the tumorigenesis process. Importantly, to determine that the specificity of the ΔNp73 effectors was non‐related to the inactivation of p53, we also confirmed their effector role in p53^−/−^cells.

The use of high accuracy and sensitivity mass spectrometry combined with SILAC labeling together with the use of antibody microarrays for the comparison of the conditioned medium from ∆Np73 and Mock stably transfected cells enabled the discovery of a large number of differentially secreted effector proteins associated to ∆Np73. Remarkably, about 76.5% of the dysregulated proteins were previously identified in the exosome content according to ExoCarta database. As ∆Np73 has been also found in the exosome of CRC cells and in the blood of CRC patients, and ∆Np73 encapsulated in exosomes has been shown to enhance tumor progression [37], we therefore hypothesize that ∆Np73 protein effectors might also help to contribute to the metastatic process or to help forming the premetastatic niche.

In this sense, we here present original data regarding the impact of ΔNp73 in metastatic cell homing and growth in lungs *in vivo* and in blood and lymphatic vessels formation in colon cancer and identified its specific effectors implicated in these processes, BDNF and the putative EMAP‐II‐VEGFC‐VEGFR3 axis. EMAP‐II is a proinflammatory cytokine with antiangiogenic activities, which can suppresses primary and metastatic tumor growth probably due to its ability to compete with VEGFs for the VEGF receptors, thus, interfering with VEGF signaling [38]. The diminished expression of EMAP‐II and the upregulation of VEGF‐C and VEGFR3 triggered by ΔNp73 overexpression along with the functional experiments in HUVEC and HLEC primary cells support the implication of this isoform in the formation of both blood and lymphatic vessels. ΔNp73 has been previously involved in the formation of blood vessels, mainly through the modulation of HIF, VEGF, and TFGβ proteins [39, 40]. To our knowledge, the implication of ΔNp73 in lymphangiogenesis has not been previously addressed. Stantic et al. [41] identified the modulation of VEGF‐C and other proangiogenic chemokines such as CCL2, CXCL1, and CXCL2 in wild type and ΔNp73^−/−^ and TAp73^−/−^primary mouse embryonic fibroblast transformed with E1A and H‐RAS^v12^ and in tumors developed in athymic nude mice subcutaneous injected with these cells. The authors robustly support the opposite functions of both TP73 variants in angiogenesis in a HIF1‐α dependent manner and identified angiogenesis and hypoxia signatures in breast cancer patients with high ΔNp73 levels. In addition to its classic consideration as a lymphangiogenic factor through activation of VEGF‐R3, VEGF‐C has also been reported to induce angiogenesis through VEGF‐R2. Our data do not rule out the implication of ΔNp73 in both axis, VEGF‐C/VEGF‐R3 and VEGF‐C/VEGF‐R2 as Stantic et al. discussed and supports the authors' results.

Noticeable, EMAP‐II is currently being used in clinical trials in different tumor types in combination with other drugs [42, 43]. In this context, ΔNp73 status may be tested as a good predictor to EMAP‐II response since its levels can be analyzed in the clinical setting in a simple qPCR. In addition, we have identified BDNF as a new effector of this isoform. BDNF has been implicated in angiogenesis through the regulation of the VEGF‐HIFs axis in different cancers [44, 45, 46]. Its participation in lymphatic vessel formation has not been reported. Here we report that the promotion of angiogenesis and lymphangiogenesis by ∆Np73 should be supported by its BDNF and EMAP‐II effectors. Taken together, our results support the idea that ΔNp73 may be a significant regulator of the formation of new vascular and lymphatic vessels during tumor spread through the modulation of different target effectors.

We next proceeded to measure these effectors in the plasma of CRC individuals and controls to evaluate their diagnostic performance. Remarkably, BDNF quantitation in plasma was observed as a candidate marker to become transferred to clinics, since it reaches AUC values up to 96.9%. Interestingly, BDNF showed a high value as early diagnostic marker of CRC since patients carrying premalignant lesions also possessed high BDNF levels in contrast to the controls of the study. However, this association presents some limitations that must be acknowledged. Because the results are based on a single study dataset, they should be reanalyzed for confirmation of our results using much larger numbers of patients with similar distributions between groups.

Moreover, other interesting effectors found to be upregulated by the overexpression of ∆Np73 were related to metabolic processes (LDHB, Aspartate aminotransferase, GLO1, PFAS, and PYGL). Optimal glucose supply to glycolytic cancer cells is a common hallmark of cancer. Among the upregulated effectors, LDHB and PYGL are associated to this supply [47, 48]. The metabolic switch from oxidative phosphorylation to aerobic glycolysis provides intermediates for cell growth and division and it is regulated by both oncogenes and tumor suppressor genes. In this sense, the control of metabolism link has been previously reported for the p53 family [49, 50]. Although TAp73 has been associated to different metabolic pathways [51], the link of ∆Np73 remained to be explored. Here, we shed some light on this process through the characterization of the ∆Np73 effectors.

Furthermore, other proteins altered by the overexpression of ∆Np73 revealed a role in cell migration (CXCR4 and S1PR1) [52, 53], and/or invasion (QSOX1) [54]. These data are supported by our functional assays monitoring these processes in HCT116 mock and ∆Np73 stably transfected cells and in our *in vivo* approach (Fig. 4). Moreover, 20% of the dysregulated proteins were related to cell adhesion (Moesin, calreticulin, sorcin, DSG2, DSC3, LAMC2, LGALS3BP, Talin‐1, CDH3, LGASLS1, PTPRF, PDCD6IP, and COL6A1). The role of cell adhesion molecules in the progression of CRC and the development of liver metastasis suggests, besides the oncogenic properties of ∆Np73, a pro‐oncogenic and pro‐metastatic property for its effectors. Undeniably, the description of new, specific oncogenic roles for ΔNp73 will help us to clarify its function in cancer either encapsulated in exosomes [37], or released to the microenvironment of the tumor. Collectively, the knowledge of proteins modulated by ∆Np73 could be useful to advance in the knowledge of CRC, CRC metastasis, and may have important implications in the selection of personalized therapy for CRC patients [31].

## 5 Conclusion

In conclusion, this unbiased proteome‐wide approach of the secretome of ΔNp73‐ and Mock‐stably transfected cells identified a large number of novel effectors of the ΔNp73 oncogenic modulator in CRC that seem to play a major role in tumor formation, progression and metastasis of CRC, and exhibit potential as diagnostic biomarkers of the disease. Further studies must be performed to determine whether the identified effectors might become new targets for therapeutic intervention in CRC.

## Conflict of interest

The authors declare no conflict of interest.

## Author contributions

MG‐A is involved in formal analysis, investigation, methodology, writing, review and editing manuscript. JR‐C is involved in formal analysis, investigation, methodology, writing, review and editing manuscript. CSM is involved in formal analysis and methodology. CP is involved in data curation, resources and review and editing manuscript. MJF‐A is involved in data curation, resources and review and editing manuscript. DP‐M is involved in formal analysis, methodology and resources. DV is involved in data curation, resources and review and editing manuscript. AM‐C is involved in formal analysis, methodology and resources. GS‐F is involved in methodology and resources. M‐ÁC is involved in data curation, resources and review and editing manuscript. MG‐C is involved in methodology and resources. NR is involved in data curation, resources and review and editing manuscript. AG‐A is involved in formal analysis, methodology, resources and review and editing manuscript. RB is involved in conceptualization, data curation, formal analysis, investigation, methodology, resources, validation, writing, review and editing manuscript. GD is involved in conceptualization, data curation, formal analysis, investigation, methodology, resources, validation, writing, review and editing manuscript.

### Peer Review

The peer review history for this article is available at https://publons.com/publon/10.1002/1878‐0261.13228.

## Supporting information


**Fig. S1.** Schematic design of the in‐depth proteomic analysis of ΔNp73 secretome effectors.

**Fig. S2.** Identification of protein interactions between the identified dysregulated proteins by the database STRING.

**Fig. S3.** Western blot of BDNF and EMAP‐II and ELISAs of VEGFC and VEGFR‐3.

**Fig. S4.** Metastasic potential of ΔNp73.

**Table S1.** Antibodies and primers used for experimental work.

**Table S2.** Clinical and pathological information of patient's serum samples used in ELISA experiments.

**Table S3.** Identified and quantified proteins and peptides in the SILAC experiment.

## Data Availability

All the data that support the findings of this study are available upon request.
