# Supplementary material for: In‐depth proteomics characterization of ∆Np73 effectors identifies key proteins with diagnostic potential implicated in lymphangiogenesis, vasculogenesis and metastasis in colorectal cancer
Source: Mol Oncol. 2022 Jun 7;16(14):2672–92. doi: 10.1002/1878-0261.13228 (PMC9298678; doi:10.1002/1878-0261.13228)
Supplement: Supplementary file 1 — Fig. S1. Schematic design of the in‐depth proteomic analysis of ΔNp73 secretome effectors. [file MOL2-16-2672-s005.pdf]

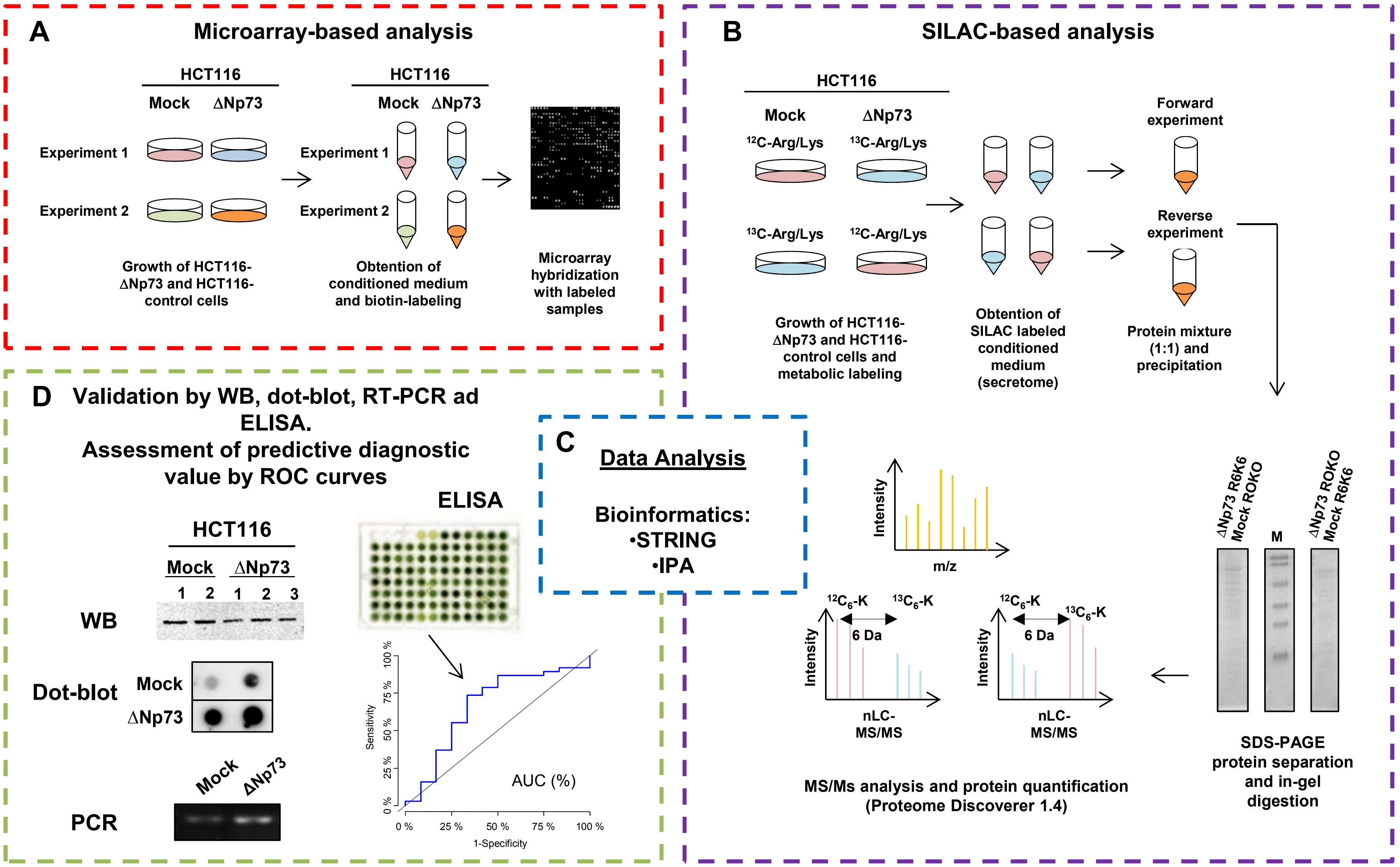

Supplementary Fig. S1

Supplementary Fig. S1.

Schematic design of the in-depth proteomic analysis of ΔNp73 secretome effectors.Microarray- (**A**) and SILAC-based (**B**) proteomic analyses were performed in parallel with HCT116-ΔNp73 and mock cells to get further insights into secretome alterations due to the overexpression of ΔNp73.For high-density antibody microarrays, cells were grown in duplicate to have biological replicates of the conditioned medium. For SILAC quantitative proteomic analysis, cells were separately cultured in both light- and heavy-labeled DMEM medium to accomplish forward and reverse experiments. Conditioned medium (secretome) was centrifuged at 250 x *g* to remove cell debris, and protein concentration quantified. Then, each cell type with different labeling was 1:1 mixed and precipitated. Proteins were separated by SDS-PAGE, cut in 10 slices and in-gel digested with trypsin prior to nano-LC-MS/MS analysis in a LTQ-Orbitrap Velos mass spectrometer to identify and quantify differentially-released proteins in the secretome of HCT116-ΔNp73 cells. Data analysis for both methodologies was carried out using different bioinformatic approaches (**C**). Validation by WB, dot-blot and semiquantitative PCR and assessment by ELISA of the predictive diagnostic value of selected candidates was carried out (**D**).
