# Supplementary material for: In‐depth proteomics characterization of ∆Np73 effectors identifies key proteins with diagnostic potential implicated in lymphangiogenesis, vasculogenesis and metastasis in colorectal cancer
Source: Mol Oncol. 2022 Jun 7;16(14):2672–92. doi: 10.1002/1878-0261.13228 (PMC9298678; doi:10.1002/1878-0261.13228)
Supplement: Supplementary file 2 — Fig. S2. Identification of protein interactions between the identified dysregulated proteins by the database STRING. [file MOL2-16-2672-s001.pdf]

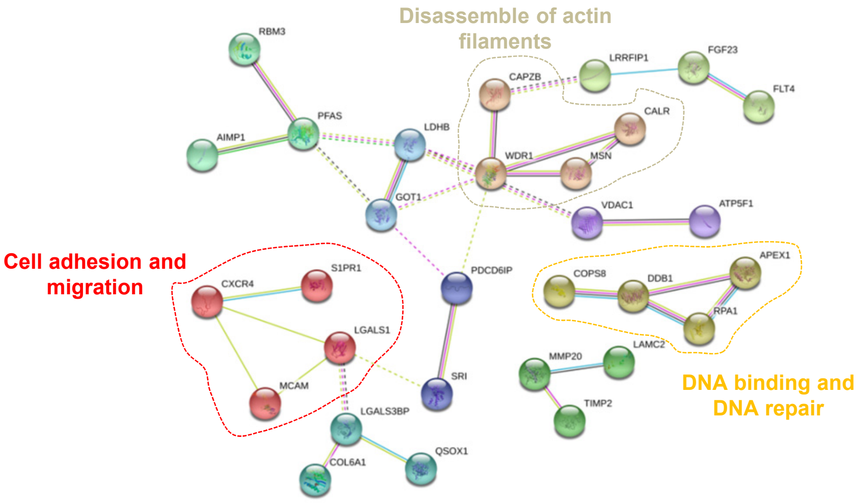

Supplementary Fig. S2

Supplementary Fig. S2.

Identification of protein interactions between the identified dysregulated proteins due to ΔNp73 over-expression in HCT116 CRC cells. The STRING data base showed a total of 7 clusters with at least three proteins. Clusters with more than three proteins were related to cell adhesion and migration, disassembly of actin filaments, and DNA binding and repair.
