# Supplementary material for: In‐depth proteomics characterization of ∆Np73 effectors identifies key proteins with diagnostic potential implicated in lymphangiogenesis, vasculogenesis and metastasis in colorectal cancer
Source: Mol Oncol. 2022 Jun 7;16(14):2672–92. doi: 10.1002/1878-0261.13228 (PMC9298678; doi:10.1002/1878-0261.13228)
Supplement: Supplementary file 3 — Fig. S3. Western blot of BDNF and EMAP‐II and ELISAs of VEGFC and VEGFR‐3. [file MOL2-16-2672-s006.pdf]

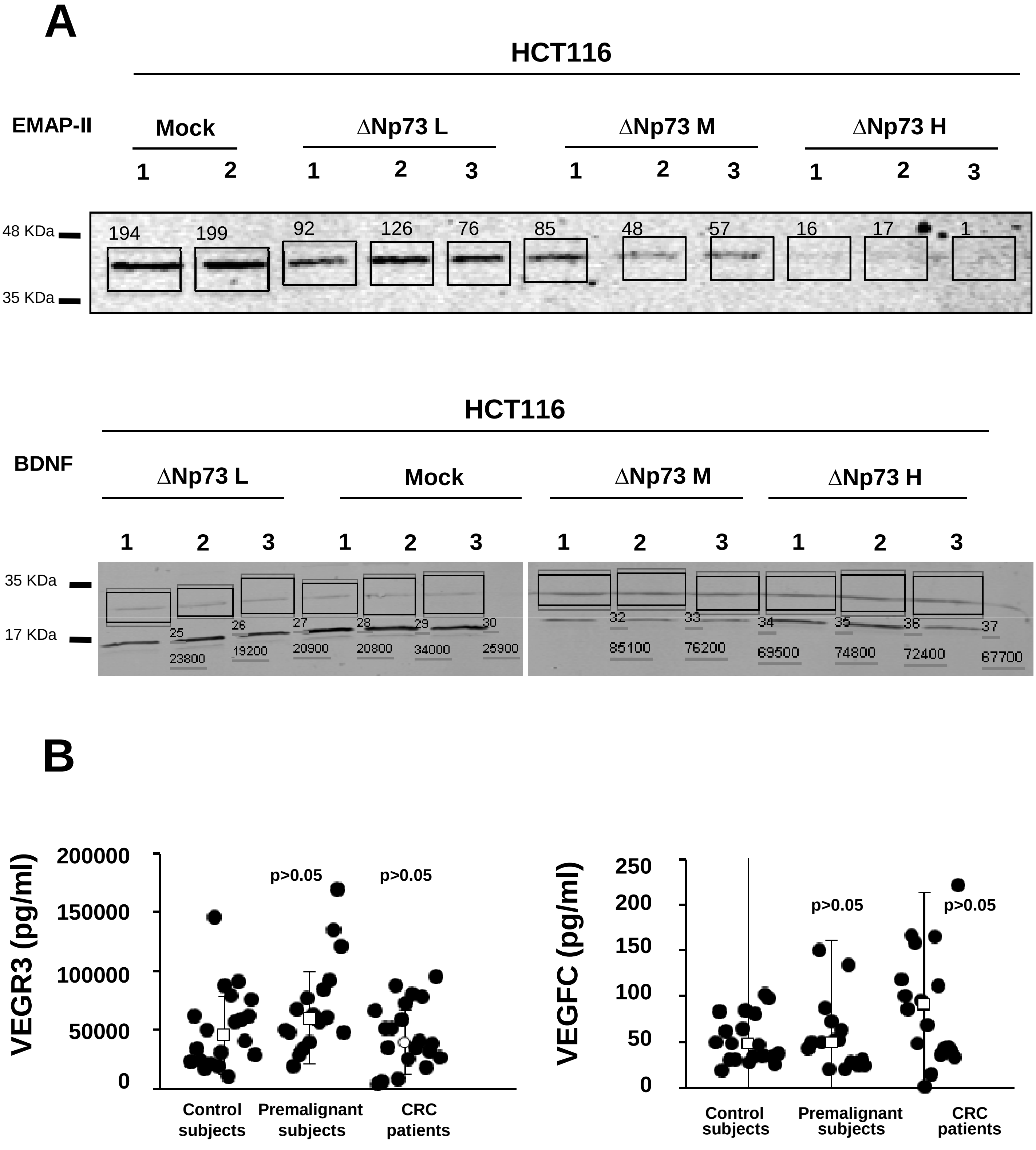

Supplementary Fig. S3.

Supplementary Fig. S3.

A. WB of EMAP-II and BDNF. L, low; M, medium; H, high are referred to the ΔNp73 levels after cell sorting of HCT116 CRC cells, where a decrease or increase expression of EMAP-II and BDNF, respectively, is observed according to the increased expression levels of ΔNp73. Squares show the quantification with the Odissey equipment (LI-COR, Rockland) represented as bar-graph in Figure 2. **B**. Evaluation of the plasma biomarker potential of VEGFR3 and VEGF. Quantification of VEGFR3 and VEGFC was performed using commercially available ELISAs and sera from control subjects, premalignant individuals, and CRC patients. Plasma levels of VEGFC and VEGFR3 were obtained for control, premalignant and CRC patients samples ($p>0.05$). Determination of their value as discriminating plasma biomarkers between control individuals and pathological subjects was not significant.
