# Supplementary material for: In‐depth proteomics characterization of ∆Np73 effectors identifies key proteins with diagnostic potential implicated in lymphangiogenesis, vasculogenesis and metastasis in colorectal cancer
Source: Mol Oncol. 2022 Jun 7;16(14):2672–92. doi: 10.1002/1878-0261.13228 (PMC9298678; doi:10.1002/1878-0261.13228)
Supplement: Supplementary file 4 — Fig. S4. Metastasic potential of ΔNp73. [file MOL2-16-2672-s007.pdf]

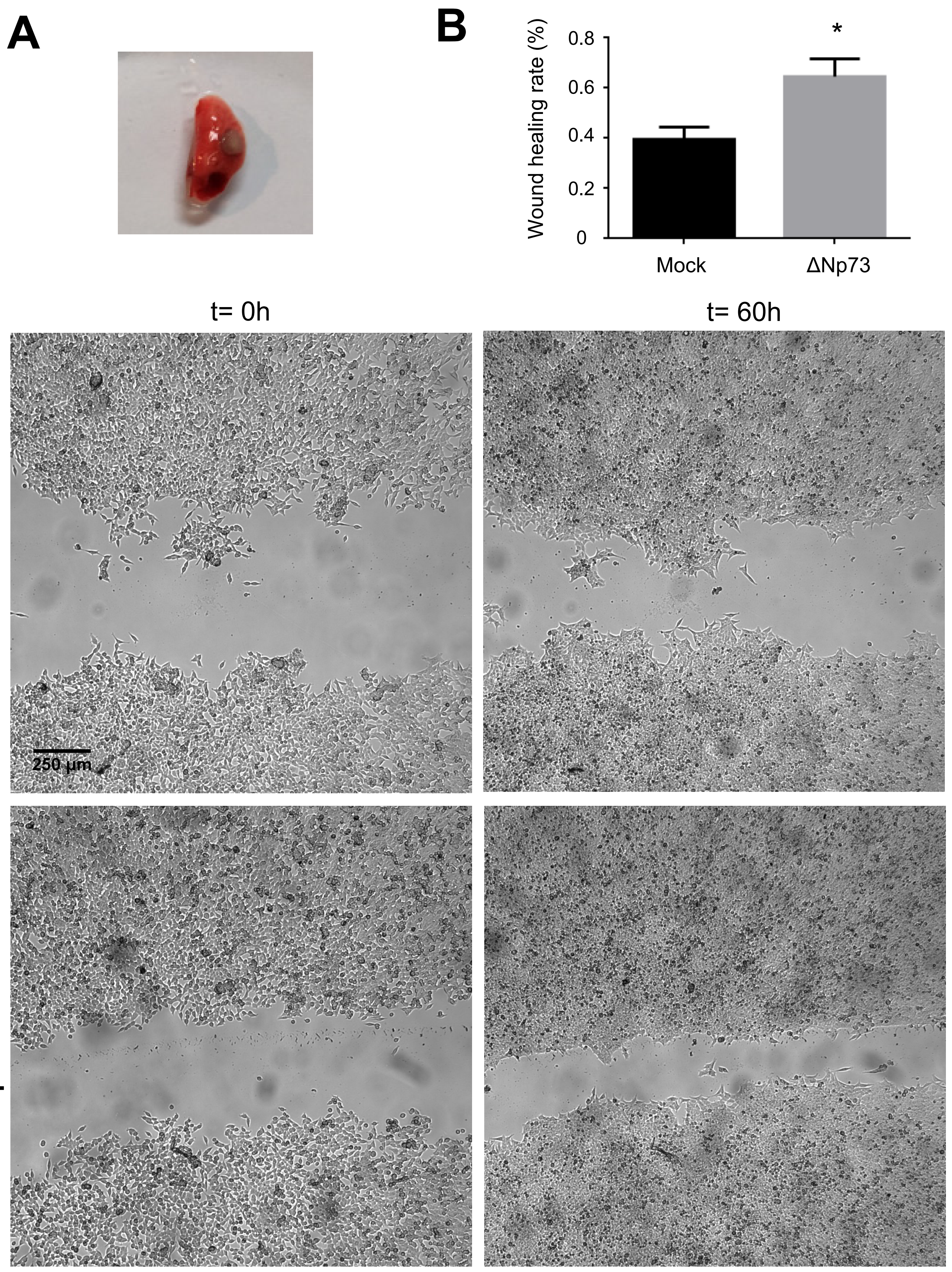

Supplementary Fig. S4.

Supplementary Fig. S4.

Metastatic potential of ΔNp73. **A,** A representative lung metastasis in a mouse systemically injected with HCT116-ΔNp73 cells is shown. Lung metastasis were observed in 3 out of 10 mice injected with HCT116-ΔNp73 cells versus none of the control mice. **B,** ΔNp73 promotes migration of HCT116 cells on the wound healing assay. Data represent speed closure mean values ± SD. $n = 5$; *$p<0.05$.
